# Severe Impact and Subsequent Recovery of a Coral Assemblage following the 1997–8 El Niño Event: A 17-Year Study from Bahia, Brazil

**DOI:** 10.1371/journal.pone.0065073

**Published:** 2013-05-31

**Authors:** Francisco Kelmo, Martin J. Attrill

**Affiliations:** 1 Instituto de Biologia, Universidade Federal da Bahia, Salvador, Bahia, Brazil; 2 Marine Institute, Plymouth University, Plymouth, Devon, United Kingdom; Institute of Botany, Czech Academy of Sciences, Czech Republic

## Abstract

The coral reefs of northern Bahia evolved in isolation from other Atlantic systems and under conditions of high environmental stress, particularly high turbidity. We have monitored the scleractinian assemblage of four shallow bank reefs (Praia do Forte, Itacimirim, Guarajuba and Abai) annually for 17 years since 1995, collecting quantitative data on diversity and density of coral colonies. As the sampling period included the 1997–8 El Niño event, the most severe on record, for the first time these results allow a quantitative assessment of the long-term impact of this major environmental stressor on such a coral assemblage. After El Niño, most species showed significantly reduced densities of colonies, this decline occurring for the subsequent two years without evidence of any new settlement until 2001. From 2000 to 2007 the species *Porites astreoides* went unrecorded. Recovery was slow, and multivariate analysis revealed that assemblages had not returned to the pre-El Niño state until 2011. It therefore took 13 years for full recovery of the coral assemblage to occur, which has consequences for reef systems if such El-Niño events become more frequent in the future.

## Introduction

The 1997–8 El Niño Southern Oscillation (hereafter ENSO) was the most extensive global ENSO event [1], [2], [3] and was associated with record global high seawater temperatures [4], [5], [6], [7], other ENSO events having had a clear impacts regionally [8], [9], but not at such a severe global scale. The evolution of ocean temperature anomalies in the Southern Hemisphere began several months earlier than the envelope of prior ENSO events and had exceptional amplitude, lasting for approximately 18 months [10]. This event followed a similar pattern in terms of the timing of its maximum development [11] and thus was similar in magnitude and duration to the 1982–3 [12] and 1986–7 events. However, it had differential effects throughout the Brazilian region [13] with increased rainfall and flooding experienced by the southernmost and central states, whilst northeastern states experienced devastating droughts [14] because of abnormally reduced rainfall in the region. Coral reefs are considered vulnerable to tipping points because of their narrow thermal and water quality tolerances, heavy reliance on key framework species and high susceptibility to nutrient runoff and eutrophication [15]. The large-scale effects during 1982–3 and 1986–7 were a tipping point for coral reefs (e.g. [16], [17]), but the most severe 1997–8 ENSO event had a more extensive impact globally on coral reefs [18], [19], [20], [21], the climatic features linked to this event causing severe coral bleaching and mortality in Panama and Ecuador [10], Indian ocean [22], Arabian Gulf [23], tropical Pacific [24], South Africa [25] and Brazil [26].

The coral reefs from northern Bahia, Brazil, have evolved and developed under natural conditions of high temperatures and high turbidity [27]. Together with the likely impact of the Amazon biogeographical barrier [28], [29], these environmental stressors have prevented the colonization of large numbers of coral taxa that are common elsewhere in the Atlantic, resulting in the Brazilian reefs having a comparatively low diversity coral fauna distinct from that of the Caribbean. Additionally, the scleractinians recorded from the northern Bahian reefs therefore have a relatively narrow geographical and environmental range and almost 40% of the species are endemic to Brazilian waters.

A long-term monitoring of these coral reefs was initiated in 1995 along northern coast of the State of Bahia, the main objective being to record temporal and spatial patterns of biodiversity and community structure of the reef-associated biota on four reef systems along this coast. The sampling period therefore included the 1997–8 ENSO event allowing, for the first time, a quantitative assessment of the long-term impact of this major environmental stressor on the biodiversity of the scleractinian assemblage and any subsequent recovery trends.

Considering that any impact on scleractinian corals has major repercussions for the reef ecosystem [30], [31], this paper reports the results of the ongoing long-term study on the scleractinian assemblage from the shallow-bank reefs of northern Bahia, using data collected over the last 17 years. We describe for the first time how the individual scleractinian coral species and the overall coral assemblage have responded to the stress imposed by the 1997–8 ENSO event, and document the time required for recovery, giving a clear indication of the temporal scale of such climatic impacts.

## Methods

### Study Area

**Figure 1:**
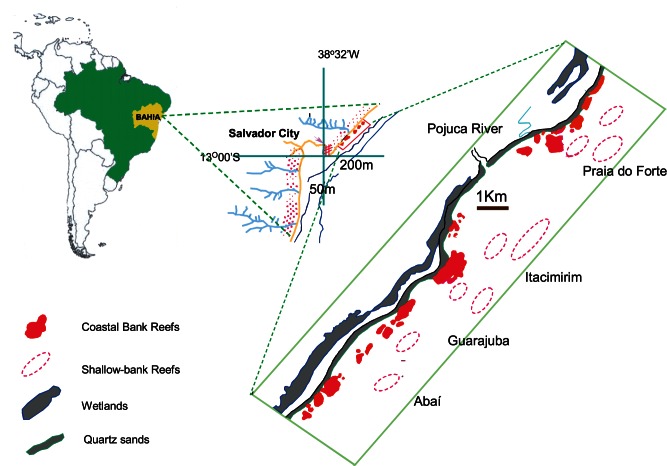
Location map showing the coral reefs of Northern Bahia Region (After Leão et al., 1997).

The four studied reefs (termed Abaí, Guarajuba, Itacimirim and Praia do Forte), exist on the narrowest part of the Eastern Brazilian Continental Shelf (average width 15 km between the Sao Francisco and Doce Rivers) and extend 20 km. The reefs of interest (Fig. 1) are Abai (12°40′04′′S/38°04′47′′W), Guarajuba (12°39′22′′S/38°03′18′′W), Itacimirim (12°37′20′′S/38°01′40′′W) and Praia do Forte (12°34′42′′S/37°58′59′′W). They are complex elongated structures varying from 500 to 1,800 m in length, from 400 to 500 m in width and in water depths between 10 and 40 m. The Pojuca River discharges near the reefs of interest; the river’s mean annual flow of 32 m^3^/s was reduced to 20 m^3^/s during the 1997–8 ENSO. The reefs are made up mainly of discrete coral heads that have developed either on rock outcrops of various ages or on lines of Holocene beachrock [32]. Their lateral contour is irregular, sometimes presenting well-developed, spur-and-groove systems on the fore-reef side, while the back-reef is usually less irregular (see [33] for details).

The coastal belt of the State of Bahia has a tropical humid climate. Annual average rainfall ranges between 1,300 mm in the north of the study area to 1,900 mm around Salvador City to the south, with no marked seasonal rainfall pattern. Average daily air temperatures range from 23° (winter) to 28°C (summer), with mean daily sea-surface temperatures ranging from 25° (winter) to 28°C (summer); the maximum SST occurs between December and February each year. Annual average salinity varies little (35–36), although within reef-top shallow pools, salinity can range from 35 to 39. The pH of seawater varies only between 8.1 and 8.2, with no clear seasonal patterns (see [34]). The coast is influenced by winds arising from the NE and E during the spring-summer, and winds coming from the SE and E during the autumn-winter season. Moreover, during the autumn-winter period, the winds arising from the SSE, associated with the periodic advance of the Atlantic Polar Front, reinforce the trade winds from the SE [35]. This pattern of wind circulation is disrupted by the quasi-cyclic environmental phenomenon known as the El Niño/La Niña and includes changes in the Atlantic Polar Front [35], with several climatic perturbations recorded [14], [36].

### Sampling

We collected density data on the scleractinian community from the four shallow bank reefs annually (between April and May) from 1995 to 2011 by SCUBA diving. Quantitative samples of the coral colonies were taken with 35 1m^2^ quadrats positioned haphazardly on each reef (depths 10–20 m) giving a total of 140 quadrats per year, and 2,380 in total over the survey period. The majority of hard coral in Bahia is present as comparatively small, isolated coral heads rather than extensive reefs, so individual colonies would be easily enumerated using this quadrat size. All corals (live and dead colonies) were counted *in situ* and any visual bleaching of the colony documented as normal, fully or partially bleached. We annotated data on color and colony size in the field along with photographic records. No specific permits were required for the described field studies as we did not need to remove any biological material from the reefs. No specific permissions were required due to the fact that this was an entirely field based study with all data being recorded on site through the in-situ identification and counting of coral colonies. The location is not privately-owned or protected in any way, as the beaches surveyed are public spaces. We did not kill or damage any of the studied organisms. These were just counted, so our methods represent no threat to the species we assessed and none is, as yet, endangered. Relative abundance analysis was assessed using the progressive scale proposed by Peixinho and Peso-Aguiar [37], resulting in density values per m^2^.

Our environmental data were from two sources: direct measurements from the sites during the survey period and data obtained from the Brazilian Meteorological Office (INMET website. http://www.inmet.gov.br/portal/index.php?r=home/page&page=rede_estacoes_conv_graf. Accessed 2013 Mar 20). As it was not logistically possible to sample water over the whole year from the site, the wider Meteorological Office data were used to look at annual patterns. Wide-scale environmental parameters for the survey area (sea surface temperature, solar irradiance, air temperature, rainfall, and cloud cover) were obtained from the INMET: these data are collected three times a day and the values presented in this paper represent the annual period around sampling. INMET data are classified internationally in ISO9001. We recorded local physicochemical data (seawater temperature, salinity, pH, and turbidity) across the four reef sites (10 replicates/reef giving 40 measurements spread over the sampling period April/May)], which was considered adequate to allow comparison between years (see 27). Temperature, salinity, and pH were recorded using a YSI63 (Yellow Spring Industries) electronic field meter (1995–2000), while turbidity was assessed using a Secchi disk, all deployed from a boat.

Since 2001 we also recorded turbidity and other local data using a Multiparameter Water Quality Meter (U5210); however, for reasons of uniformity we present the same type of measurement throughout the years. Depth (below low tide) was recorded at each sampling station.

Previous analysis has demonstrated no significant effect of location on community data collected from within each reef [38], so the significance of differences between years was assessed using either ANOVA [log (x+1) transformed coral densities, normally distributed environmental variables] or Kruskall-Wallis (non-normal environmental data, no transformation) tests (α = 0.05). Post-hoc pairwise comparisons were undertaken using either Tukey-Kramer or Dunn’s multiple comparison tests.

For multivariate analysis, the coral density data were normalized and log(x+1)-transformed in order to reduce the influence of dominant species [39]. A triangular matrix of similarities between samples was computed using the similarity coefficient of Bray and Curtis [40]. The similarity matrix was subjected to ordination analyses using the PRIMER (Plymouth Routines in Multivariate Ecological Research) package [41]. Ordination was by non-metric multidimensional scaling (MDS). We examined the contribution of species to dissimilarities between the groupings observed in the ordination analyses using the SIMPER procedure (similarity percentages; [42]). We used the BIOENV method [43] to investigate the relationship between environmental variables (pre- and post- ENSO) and the coral community data. This method is used as an exploratory tool in ways analogous to multiple regression [43] and correlates the similarity matrix derived for the coral communities with an equivalent for the suite of environmental measurements taken at each site. Results are expressed as a Spearmans correlation coefficient (r), ranked in the order of which single variable or combination of variables best explains the observed community patterns [43]. The results (maximum of 1) indicate the proportion of variance in the community data explained by these environmental variables (see [43] for full details). We tested for significance difference in coral community composition between years using Two-way crossed ANOSIM [44].

## Results

The 1997–8 ENSO had a significant influence on most of the measured environmental parameters (Table 1). Mean air and seawater temperatures and hours of sunlight increased significantly in 1998 compared with all other years, the latter due to lower cloud cover. Rainfall was significantly lower during ENSO conditions and this resulted in reduced freshwater and sediment outflow from the local rivers (the mean annual discharge of the São Francisco River was reduced from 32,980 to 1,768 m^3^s^−1^ and that of Doce River from 80.5 to 50.2 m^3^s^−1^) and, thus, significantly clearer water. 1998 was therefore characterized by warmer air and sea temperatures, reduced cloud cover and rainfall, higher incoming solar radiation, and reduced turbidity (due mainly to reduced river runoff following decreased precipitation). Similar, but not so intense, conditions were observed in 2007 and 2010. In contrast, 1999–2000, and to a lesser extent, 1995–6 represented relatively strong La Niña conditions, as indicated by high rainfall and cloud cover (Table 1).

**Table 1:** Summary of wide-scale and locally measured physico-chemical data recorded from the shallow bank Reefs throughout the sampling period.

	Wide-scale parameter recorded (Mean value ± SE) (*n* = 120)				
Years	Sea-surfacetemperature (°C)	Sunlight irradiation(h/yr)	Air temperature (°C)	Rainfall (mm^3^)	Cloud cover (Dec)
1995	25.6±0.05	2110±2.5	26.4±0.11	1410±2.2	4.57±0.03
1996	25.4±0.03	2080±2.5	26.2±0.05	1950±2.5	4.55±0.03
1997	25.6±0.03	2190±2.5	26.5±0.03	1350±4.8	4.52±0.03
1998	27.7±0.06	2410±7.5	28.7±0.02	1150±2.9	3.97±0.03
1999	25.5±0.13	2100±2.5	26.6±0.10	1480±2.5	4.77±0.06
2000	25.5±0.04	2050±7.1	26.5±0.04	1950±2.5	5.57±0.09
2001	25.4±0.04	2120±2.8	26.6±0.05	1420±2.0	4.89±0.06
2002	25.5±0.08	2160±2.4	26.4±0.12	1440±2.8	4.68±0.04
2003	25.7±0.03	2180±3.0	26.6±0.08	1510±2.6	4.45±0.03
2004	25.5±0.10	2200±2.8	26.7±0.09	1580±3.8	4.53±0.04
2005	25.6±0.05	2190±4.5	26.8±0.05	1600±4.5	4.42±0.02
2006	25.2±0.09	2120±3.5	26.2±0.04	1900±3.2	4.53±0.03
2007	25.5±0.06	2210±4.2	26.9±0.08	1340±4.2	4.49±0.03
2008	24.9±0.14	2100±5.1	26.3±0.08	1550±2.8	4.57±0.04
2009	25.6±0.03	2180±3.4	26.4±0.10	1460±2.6	4.77±0.05
2010	25.4±0.08	2220±4.2	26.7±0.09	1420±3.2	4.81±0.02
2011	25.5±0.06	2160±3.5	26.6±0.04	1400±3.1	4.63±0.03
	**Local parameter recorded (Mean value ± SE) (** ***n*** ** = 40)**				
**Years**	**Seawater temperature** **(bottom; °C)**	**Salinity**	**Air temperature (°C)**	**pH [H** ^−**1**^ **]**	**Turbidity (Secchi clarity, m)**
1995	25.1±0.01	36.3±0.11	26.8±0.11	8.2±0.02	1.65±0.08
1996	25.0±0.01	36.3±0.04	26.6±0.05	8.2±0.01	1.65±0.11
1997	25.4±0.01	36.4±0.17	26.9±0.03	8.3±0.02	1.85±0.06
1998	26.9±0.01	36.7±0.09	29.1±0.02	8.2±0.02	2.47±0.05
1999	25.2±0.07	36.5±0.16	26.9±0.09	8.3±0.02	1.62±0.04
2000	25.1±0.09	36.5±0.15	26.8±0.06	8.2±0.03	1.57±0.08
2001	25.4±0.01	36.4±0.01	26.7±0.08	8.2±0.01	1.58±0.01
2002	25.6±0.01	36.4±0.01	26.6±0.07	8.2±0.01	1.49±0.01
2003	25.7±0.01	36.4±0.02	26.8±0.09	8.2±0.01	1.48±0.01
2004	25.6±0.01	36.4±0.02	27.0±0.10	8.3±0.01	1.53±0.01
2005	25.5±0.01	36.4±0.02	26.8±0.02	8.2±0.01	1.54±0.01
2006	25.4±0.01	36.5±0.02	26.5±0.06	8.2±0.01	1.62±0.02
2007	25.5±0.01	36.5±0.02	27.3±0.12	8.3±0.01	1.64±0.01
2008	25.6±0.01	36.4±0.02	26.5±0.07	8.5±0.01	1.52±0.02
2009	25.7±0.01	36.4±0.01	26.6±0.06	8.3±0.01	1.48±0.01
2010	25.7±0.01	36.5±0.02	27.0±0.05	8.2±0.01	1.52±0.01
2011	25.7±0.01	36.4±0.01	26.9±0.03	8.3±0.01	1.64±0.01

Local parameters recorded during the sampling months (April/May).

**Figure 2:**
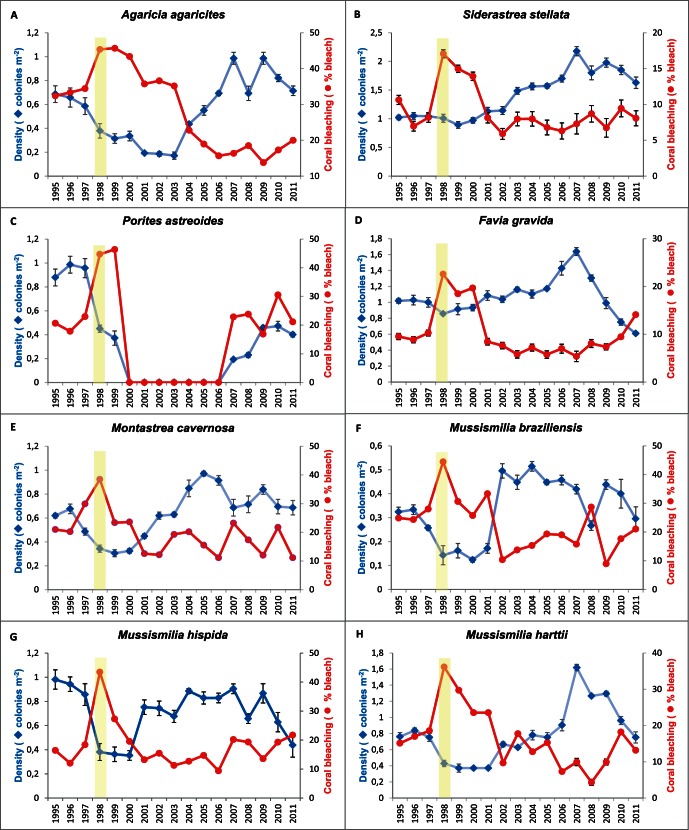
Changes in density and % of bleaching (mean ± sd) of the eight coral species recorded over a 17 year period 1995–2011 in Bahia, Brazil. Vertical coloured bar represents the time period of the 1997–8 severe El Niño;

We recorded eight species of scleractinian corals (Fig. 2a–h) from the four shallow bank reefs of northern Bahia: *Agaricia agaricites* (Linnaeus, 1758); *Favia gravida* Verrill,1868; *Montastraea cavernosa* (Linnaeus, 1766); *Mussismilia braziliensis* (Verril, 1868); *Mussismilia harttii* (Verrill, 1868); *Mussismilia hispida* (Verrill, 1901); *Porites astreoides* Lamark, 1816 and *Siderastrea stellata* Verrill, 1868. Each species was recorded from all four locations (Praia do Forte, Itacimirim, Guarajuba and Abai) during the investigation period. There was no significant variation in species composition between reefs.

We observed partially bleached corals in each year of the survey. Only the colonies of *Porites astreoides* were fully bleached between 1997–8. The percentage of bleached corals was significantly higher in 1997–8 (Fig. 2a–h) than during 1995–96 (Two-way ANOVA, F_16,527_ = 22.351, p<0.001) remaining comparatively high for the following two years (strong La Niña).

**Figure 3:**
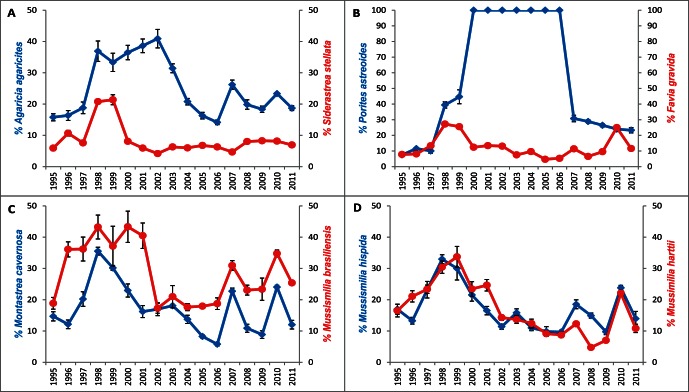
Trends over 17 years in % mortality for the eight coral species recorded from Bahia, Brazil. **a**. *A.agaricites* & *S. stellata*. **b**. *P. astreoides* & *F.gravida*. **c**. *M. cavernosa* & *M. brasiliensis*. **d**. *M. hispida* & *M.harttii*. Data expressed as % dead colonies, vertical bar represents the 1997–8 El Niño event.

The percentage of colony mortality, recorded in each year, was relatively low during pre-ENSO, but increased for all species in 1998 (Fig. 3a–d). Highest initial mortality was suffered by *M.brasiliensis* (from 18.8±1.88% in 1995 to 43.24±3.82% in 1998), followed by *P.astreoides* (7.52±0.43%–39.4±1.98%), *A.agaricites* (15.8±1.13%–36.9±3.27%) and *M.cavernosa* (14.7±1.47%–35.5±1.25%). The percentage of dead colonies decreased continuously until 2007 when we recorded increased mortality to *P.astreoides* (30.76±1.56%), *M.braziliensis* (30.95±1.51%) and *A.agaricites* (26.2±1.66%). We recorded an increase of mortality in 2010 for all species but *S.stellata.* During the whole investigation lowest mortality was experienced by *S.stellata* (5.92±0.82%–20.78±0.53%), but its recovery was evident by 2000 (only 5,95±0.41% mortality). Since then mortality has fluctuated within 10%.

A further consequence of the ENSO event was the significant reduction in coral density in 1998 (Two-way ANOVA, F_16,527_ = 6.043, p<0.0001), the decline continuing during the strong La Niña cycle in 1999–2000 (Fig. 2a–h) which resulted in local disappearance of *P.astreoides* in the year 2000; it was then absent from the reefs for seven years (see supporting information). From 2001 coral density increased continuously until 2007 to a maximum mean of 8.67±0.03 colonies m^−2^ (Fig. 2a–h). Conversely, in the same period the percentage of bleached colonies (Fig. 3a–h) decreased to values below 20% and this coincided with the reappearance of *P. astreoides* on all four reefs in 2007 after its absence. Since 2007 all eight species recorded during the pre-ENSO period have been present across the four studied reefs. Over recent years, we have documented some density reduction trends, but for most species these seem to be returning to values similar to those recorded during the pre-ENSO years of monitoring (1995–7). As of 2011, however, densities of *M. hispida, F. gravida* and *P. astreoides* are below the pre-ENSO state.

**Figure 4:**
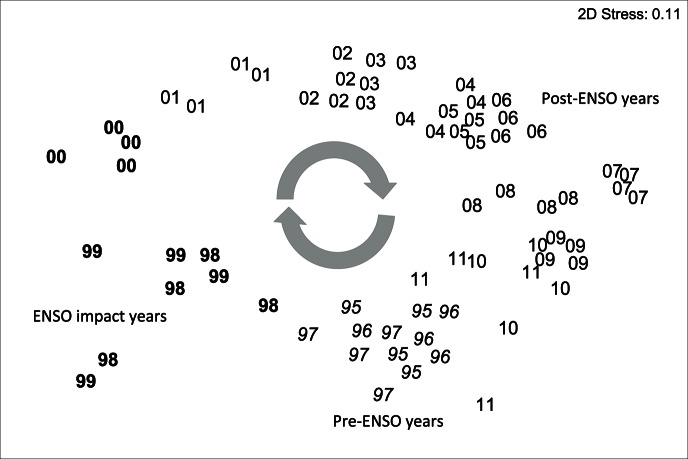
Non-metric multidimensional scaling ordination of the coral assemblage data from the four assessed shallow-bank reefs from northern Bahia (Praia do Forte, Itacimirim, Guarajuba and Abai) throughout the sampling period, 1995–2011. Arrows indicate cyclical direction of change. Codes refer to year of sampling (i.e. 00 = 2000). The nearer the data labels, the more similar the assemblages between those two samples.

MDS ordination illustrates three different periods in the progression of the studied coral assemblage (Fig. 4): (i) Years from 1995–7, i.e. pre-ENSO years [SIMPER average similarity 94.21]; (ii) Years from 1998–2000, termed ENSO-impact years [average similarity 87.00] and, (iii) Years 2001–11, post-ENSO years (average similarity [82.80]). Through this ordination analysis, it is clear that the studied coral assemblage has changed significantly over these seventeen years (two-way crossed ANOSIM, r = 0.637, p<0.01), first suffering the devastating impacts of the 1997–8 ENSO/1999–2000 La Niña events and eventually recovering from it, with little apparent influence from any other further low intensity ENSO events that have occurred since 2001. By 2011, samples were similar to those from the 1995–7 pre-ENSO period. Coral assemblage recovery from such an ENSO event therefore has taken 13 years.

Finally, BIOENV analysis indicated that variation in turbidity (r = 0.32) was the main environmental factor best explaining the coral community differences on the assessed reefs in non-ENSO years. However, a combination of turbidity (r = 0.65), mean temperature (r = 0.64), and cloud cover (r = 0.56) best explained the changes in the community in 1998, and again in 2007 and 2010.

## Discussion

The impact of the 1997–8 ENSO event on the coral reef fauna of Bahia has been well documented [45], [46], [47], [48]. Such impacts are in common with other studies [27], [49] as well as other reef systems around the globe (e.g. [50], [51]); however, there are few data on recover patterns from Brazil (but see [52 and 53).

Multivariate analysis demonstrated that the coral community from the shallow bank reefs became significantly different from 1998 onwards, compared with previous years. Considering the documented large-scale effects of the 1997–8 ENSO on coral reefs [54], it is reasonable to connect these clear changes in the coral community from 1998 onwards with this stressing event, as they are linked with differences in abiotic variables that year [increased temperature, reduced turbidity and cloud cover (Table 1)]. In addition, as there was bleaching in each studied year and diversity remained comparatively unaffected, we consider decline in density of live colonies (increase of mortality) as the main initial response of the Bahian coral community to ENSO events [38].

The decline in productivity, and subsequent reduction of the food supply following the ENSO stress period [55], leads to the disruption of many trophic links in coral reef communities [27], [55]. The effects of this nutritional deficit [56], [57], [58], [59], [60], [61], [62], [63] reduces the capacity of corals to compete favorably for space with other reef associated organisms, leading to the coral decline we documented during the 1997–8 ENSO.

Apart from *P. astreoides*, the hermatypic corals from the study reefs appeared comparatively resistant to ENSO events. There was a decrease in density of live colonies, but the response to ENSO conditions was similar for all reefs. In the past [48], comparisons between densities of bleached and non-bleached corals along the study area reflected the resistance of the Bahian corals to prolonged environmental stress. There was significant increase in the percentage of bleached colonies between 1998–2000; however, the average overall bleaching (36.5%) and subsequent mortality (33.3%) was lower than expected for such a strong ENSO event when compared with other areas such as Micronesia [64], Pacific [65] and Indian Ocean [66], [67], where the occurrence of bleaching was abnormally high.

It is important to highlight that a peculiar coralline fauna exists in the Northern Bahia shallow bank Reefs, as half of the recorded species are endemic. Some relevant aspects that increase survival rate of scleractinian corals (see [68] and references therein) includes phenotypic plasticity, which confers them tolerance to various environmental conditions and, resistance to changing habitat conditions, efficiency in removal of accumulated sediment and high energy investment to protect and reduce possible inter-specific competition for potential growth space [69]. Rapid colonisation of available substrata can be as result of higher investment in sexual reproduction and periodic release of planulae resistant to limiting resources [68], [70]. Although it is energy demanding, corals attempt to clean themselves of sediments by a combination of ciliary action and the production and sloughing off of mucus layers [71] and references therein. More detailed studies are needed, however, before it can be verified whether the size of the corallites and the ciliar mechanism of the corals may be held responsible for the settlement success of these species in south Atlantic [72], thus favoring the colonisation of turbid waters or facilitating prey capture. In addition, all species recorded in this study form massive colonies (high metabolic rates), that acclimatize more effectively to environmental changes than those with low metabolic rates [73]. This ability is partly due to these coral skeletons having a high absorbance of solar ultraviolet radiation [74], which seems to be crucial for the success of corals in the tropics. Therefore, it is likely that the coralline fauna from northern Bahia displays evolutionary modifications in behavior, morphology and physiology, enabling them to acclimate to changing climatic conditions [775]. However, despite some efforts in the last decade [76], [77], [78], [79] the taxonomy of Brazilian corals has not been fully addressed, and a detailed taxonomic review is required to verify the distinctiveness of these endemics, including data on molecular biology and ecology, endosymbionts and behavior.

As reviewed by Jokiel and Coles [80], individual coral colonies living in high temperature environments (in our study, *F.gravida* and *S.stellata* also exist in hot reef top pools), can survive and photosynthesize at temperatures a few degrees higher than their congeners in lower temperature environments [56], [12]. Depending upon the area, they tolerate sustained temperatures of 30°C for several weeks [81], [82], or 32–34°C from several days to a few weeks [83]. Therefore, according to Clausen and Roth [84], many species are able to acclimate physiologically to increased temperatures, a suggestion validated by recent studies [85], [86] as well as by the response displayed by *F. gravida* and *S. stellata*, which seemed quite resistant to the elevated temperatures recorded in northern Bahia during the ENSO period. However, even endemic species (*M.hispida* and *M.harttii*) or widely distributed [87], disease resistant [88] corals, such as *P.astreoides*, succumbed to excessive and prolonged periods of warming, such as the strong 1997–8 ENSO followed by the 1999–2000 strong La Niña-related conditions of increased rainfall and therefore increased runoff of terrestrial nutrients [34]. No significant step change in the studied community was noticed between 2001–2005 when thermal anomalies varying from 0.25°C to 0.75°C were reported for the coast of Bahia [26], nor during the 2009–10 ENSO that caused bleaching in another regions [89]. This reinforces the idea of heat-adaptability [86], [90] and that the studied corals are locally adapted to such fluctuations [27], [38]. There are evidences that exposure to high levels of photosynthetically active radiation (PAR) [91] or ultraviolet radiation (UVR) are damaging the coral community [92], [93]. Therefore, we suggest that abnormally prolonged high temperature changes [85], [90], [94] associated with increased intense exposure to solar radiation [91] and subsequent mortality should be held responsible for the significant loss of density, observed in this study.

It has also been suggested that increased water flow causes coral bleaching and all its consequences (for *Montastraea annularis*; [95], [96]), but this does not seem a contributory mechanism in Bahia as there was no significant change in water flow in the region of the studied reefs. Additionally, although fine scale data on such water quality parameters were not available, we have no indication that other factors causing coral bleaching, disease or mortality, such as pollution or viruses, were influencing Bahian reefs during the studied period. Our data, and BIOENV analysis performed in previous studies [34], [46], supports our hypothesis that the large-scale impacts of the 1997–8 ENSO on these reefs are linked with factors additional to the abnormally high seawater temperatures. These included reduced cloud cover and increased clarity of the water due to reduced land runoff, resulting in increased levels of solar radiation reaching the reefs, which has been reported to have negative effects on corals ([91], [97], [98], [99] and many others).

Finally, rapid evolution, including differentiation into new species, may occur in corals with large genetic variability, relatively short life spans, and limited back crossing with older individuals [56]. Potts and Garthwaite [100] suggested that these attributes in Caribbean species of *Porites* are responsible for their apparently rapid evolution during late Quaternary time. However, the impact on *Porites* from the change in the environment associated with the 1997–8 ENSO, followed by the strong 1999–2000 La Niña, was so severe that the entire local population died out in 2000 and the species remained unrecorded for seven years. Conversely, it has been stated that *Porites* is not as susceptible as other genera to heat-light stress-induced mortality [101], [102], and that their gene expression patterns are responsible to stress, rendering them to a consistent reliable indicator [103]. This reinforces the idea that the coral fauna from Northern Bahia, mostly endemic relics, are more locally adapted to the natural environmental stress than any other species, even the genus *Porites* that has a wide distribution across the Atlantic.

## Supporting Information

Table S1 **Total density (140 m-2) per year of the corals from the reefs of interest.**
(DOCX)
