# Supplementary material for: Severe Impact and Subsequent Recovery of a Coral Assemblage following the 1997–8 El Niño Event: A 17-Year Study from Bahia, Brazil
Source: PLoS One. 2013 May 31;8(5):e65073. doi: 10.1371/journal.pone.0065073 (PMC3669085; doi:10.1371/journal.pone.0065073)
Supplement: Table S1 — Total density (140 m-2) per year of the corals from the reefs of interest. (DOCX) [file pone.0065073.s001.docx]

**Table S1**. Total density (140 m^-2^) per year of scleractinian corals from the four shallow bank reefs of northern Bahia throughout the sampling period (1995-2011).

| **Species** | **1995** | **1996** | **1997** | **1998** | **1999** | **2000** | **2001** | **2002** | **2003** | **2004** | **2005** | **2006** | **2007** | **2008** | **2009** | **2010** | **2011** |
| --- | --- | --- | --- | --- | --- | --- | --- | --- | --- | --- | --- | --- | --- | --- | --- | --- | --- |
| *Agaricia agaricites* | 2.74 | 2.63 | 2.34 | 1.51 | 1.26 | 1.34 | 0.77 | 0.74 | 0.69 | 1.74 | 2.20 | 2.77 | 3.94 | 2.77 | 3.94 | 3.29 | 2.86 |
| *Siderastrea stellata* | 4.09 | 4.20 | 4.17 | 4.03 | 3.57 | 3.89 | 4.51 | 4.57 | 5.94 | 6.26 | 6.29 | 6.80 | 8.71 | 7.20 | 7.91 | 7.40 | 6.51 |
| *Porites astreoides* | 3.51 | 3.94 | 3.83 | 1.80 | 1.49 | 0.00 | 0.00 | 0.00 | 0.00 | 0.00 | 0.00 | 0.00 | 0.77 | 0.91 | 1.83 | 1.89 | 1.60 |
| *Favia gravida* | 4.06 | 4.20 | 4.09 | 3.46 | 3.74 | 3.63 | 4.23 | 4.17 | 4.57 | 4.54 | 4.63 | 5.57 | 6.66 | 5.31 | 4.06 | 2.91 | 2.40 |
| *Montastrea cavernosa* | 2.49 | 2.69 | 2.03 | 1.40 | 1.26 | 1.34 | 1.77 | 2.37 | 2.46 | 3.23 | 3.80 | 3.71 | 2.91 | 2.80 | 3.23 | 2.89 | 2.71 |
| *Mussismilia brasiliensis* | 1.23 | 1.31 | 1.06 | 0.60 | 0.63 | 0.49 | 0.71 | 1.91 | 1.83 | 2.00 | 1.83 | 1.86 | 1.66 | 1.14 | 1.69 | 1.71 | 1.34 |
| *Mussismilia hispida* | 4.06 | 3.91 | 3.69 | 1.74 | 1.60 | 1.46 | 2.89 | 2.89 | 2.89 | 3.46 | 3.40 | 3.20 | 3.51 | 2.77 | 3.17 | 2.74 | 2.11 |
| *Mussismilia harttii* | 2.89 | 3.31 | 3.00 | 1.83 | 1.63 | 1.40 | 1.49 | 2.57 | 2.51 | 3.23 | 3.11 | 3.57 | 6.51 | 5.14 | 5.29 | 4.03 | 3.29 |
| **TOTAL** | **25.06** | **26.20** | **24.20** | **16.37** | **15.17** | **13.54** | **16.37** | **19.23** | **20.89** | **24.46** | **25.26** | **27.49** | **34.69** | **28.06** | **31.11** | **26.86** | **22.83** |
